# L-DOPA Autoxidation: An Empirical Valence Bond Simulation of the Reactive Step

**DOI:** 10.1021/acs.jpcb.4c03002

**Published:** 2024-08-24

**Authors:** Alja Prah, Janez Mavri

**Affiliations:** † Laboratory for Computational Biochemistry and Drug Design, National Institute of Chemistry, Ljubljana 1000, Slovenia; ‡ Networking Infrastructure Centre, Jožef Stefan Institute, Ljubljana 1000, Slovenia

## Abstract

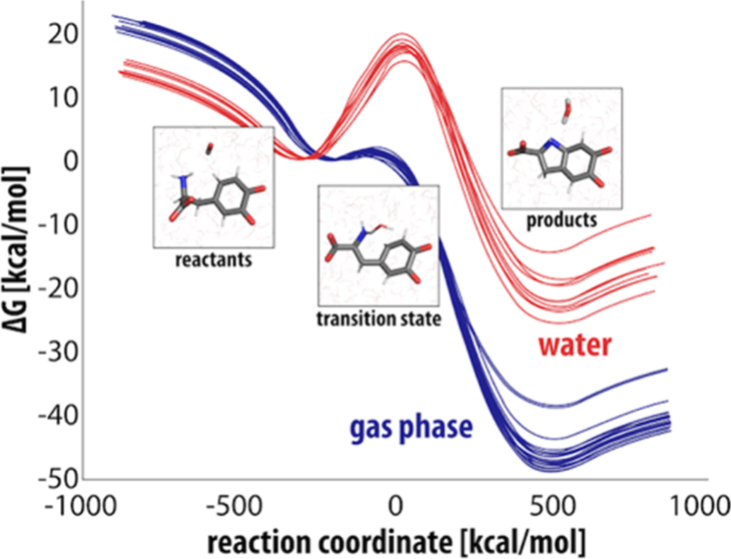

L-DOPA, or levodopa, plays an important role in the treatment of Parkinson’s disease, a debilitating neurological disorder. It acts as a precursor to dopamine, a neurotransmitter crucial for the regulation of motor functions. Administered orally, L-DOPA easily crosses the blood–brain barrier and converts into dopamine in the brain, relieving symptoms such as tremors and rigidity. However, its prolonged use can lead to complications. A significant concern with L-DOPA is its conversion to dopaquinone, a quinone metabolite that enters the redox cycle and continuously produces hydrogen peroxide. In addition, L-DOPA, which resembles tyrosine with an additional hydroxyl group, can randomly incorporate into the proteins of dopaminergic neurons and thus become an additional source of oxidative stress in Parkinson’s patients. In this study, we scrutinized the rate-limiting step of L-DOPA autoxidation in aqueous solution. The reaction we studied is an intramolecular Michael addition concerted with a proton transfer from the amino group. Using the Empirical Valence Bond (EVB) method, we computed the free energy profiles of the reaction in water. The calculated barrier of 30.93 ± 1.12 kcal/mol is in reasonable agreement with the experimental barrier of 27.55 kcal/mol. This agreement confirms the validity of the studied mechanism and demonstrates the applicability of our simulation methodology for studying the autoxidation kinetics of L-DOPA within proteins.

## 1 Introduction

L-DOPA, short for levodopa, is the drug of choice in the treatment of Parkinson’s disease, a neurodegenerative disorder that impairs motor skills. This drug acts as a precursor to dopamine, a neurotransmitter crucial for motor control and other neurological functions. Unlike dopamine, L-DOPA can cross the blood–brain barrier when administered orally. There it is converted into dopamine and replenishes the depleted levels of this neurotransmitter in the brain. Its introduction in the 1960s revolutionized Parkinson’s treatment, providing significant relief from symptoms such as tremors, rigidity, and bradykinesia. It is usually administered together with carbidopa, an inhibitor of aromatic l-amino acid decarboxylase, which prevents the peripheral metabolism of L-DOPA.

However, the path of L-DOPA into clinical practice was complicated.^1,2^ It was first synthesized by Funk^3^ and isolated from broad bean (*Vicia faba*) seeds by Gugenheim.^4^ Its therapeutic potential was demonstrated by Carlsson,^5^ whose groundbreaking work was awarded the Nobel Prize in Medicine or Physiology in 2000. Other pioneers such as Hornykiewicz,^6^ Cotizas^7^ and Yahr^8^ played an important role in bringing L-DOPA from bench to bedside.^9^ While L-DOPA is very effective in managing Parkinson’s symptoms, it can also cause various side effects such as dyskinesias and psychiatric disorders such as hallucinations and mood swings. L-DOPA is either decarboxylated to dopamine or oxidized to its quinone form dopaquinone, which significantly increases oxidative stress via two different mechanisms: (a) autoxidation or (b) incorporation into proteins. This second effect went unnoticed for a long time, or at least was not mentioned in the literature until recently.^10^ Dopamine, on the other hand, is susceptible to rapid oxidation to its quinone form. Both dopamine and dopaquinone react with a hydroxide ion, producing bicyclic leukodopachrome and reactive oxygen species (ROS) as byproducts. The latter step is rate-limiting for dopamine and most likely also for dopaquinone. Prolonged exposure of the central nervous system to ROS is highly detrimental and can damage the neural membranes and induce amyloid plaque formation, both of which lead to neurodegeneration. Therefore, strategies to mitigate oxidative stress in the treatment of Parkinson’s often involve combining L-DOPA with antioxidants or developing adjunct therapies that target ROS production pathways. Understanding and managing oxidative stress is crucial for the optimization of therapeutic benefits of L-DOPA while minimizing its potentially harmful effects.

**Figure 1:**
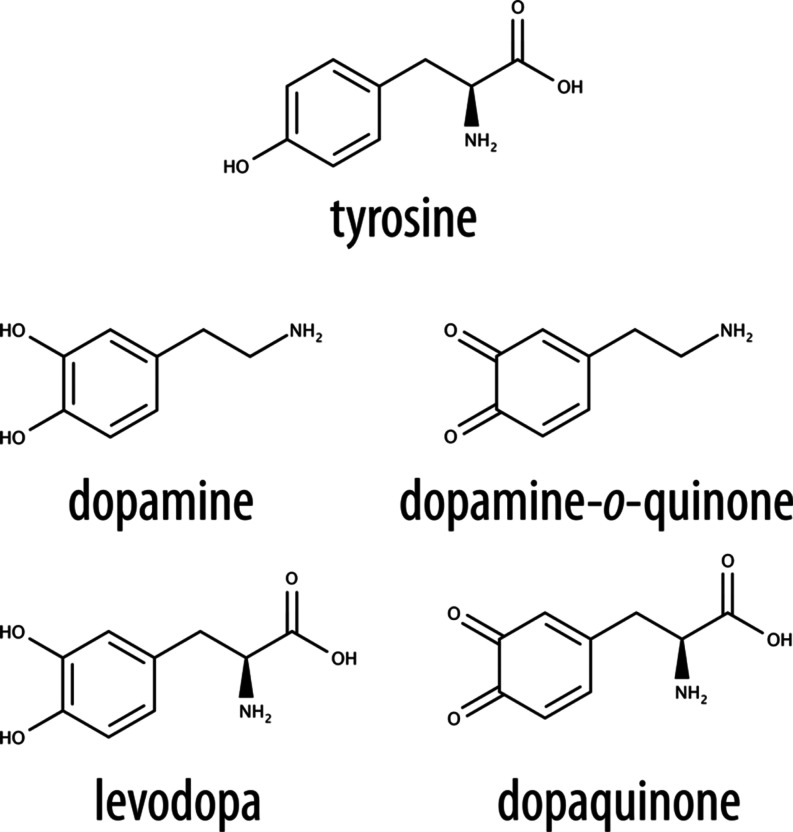
Structures of tyrosine, dopamine, dopamine-*o*-quinone, levodopa (L-DOPA) and dopaquinone.

L-DOPA shares structural similarities with tyrosine and dopamine (Figure 1) and is even present in shellfish proteins. The resulting oxidative stress may explain why shellfish adhere tightly to underwater surfaces like the bottom of ship hulls.^11−13^

**Figure 2:**
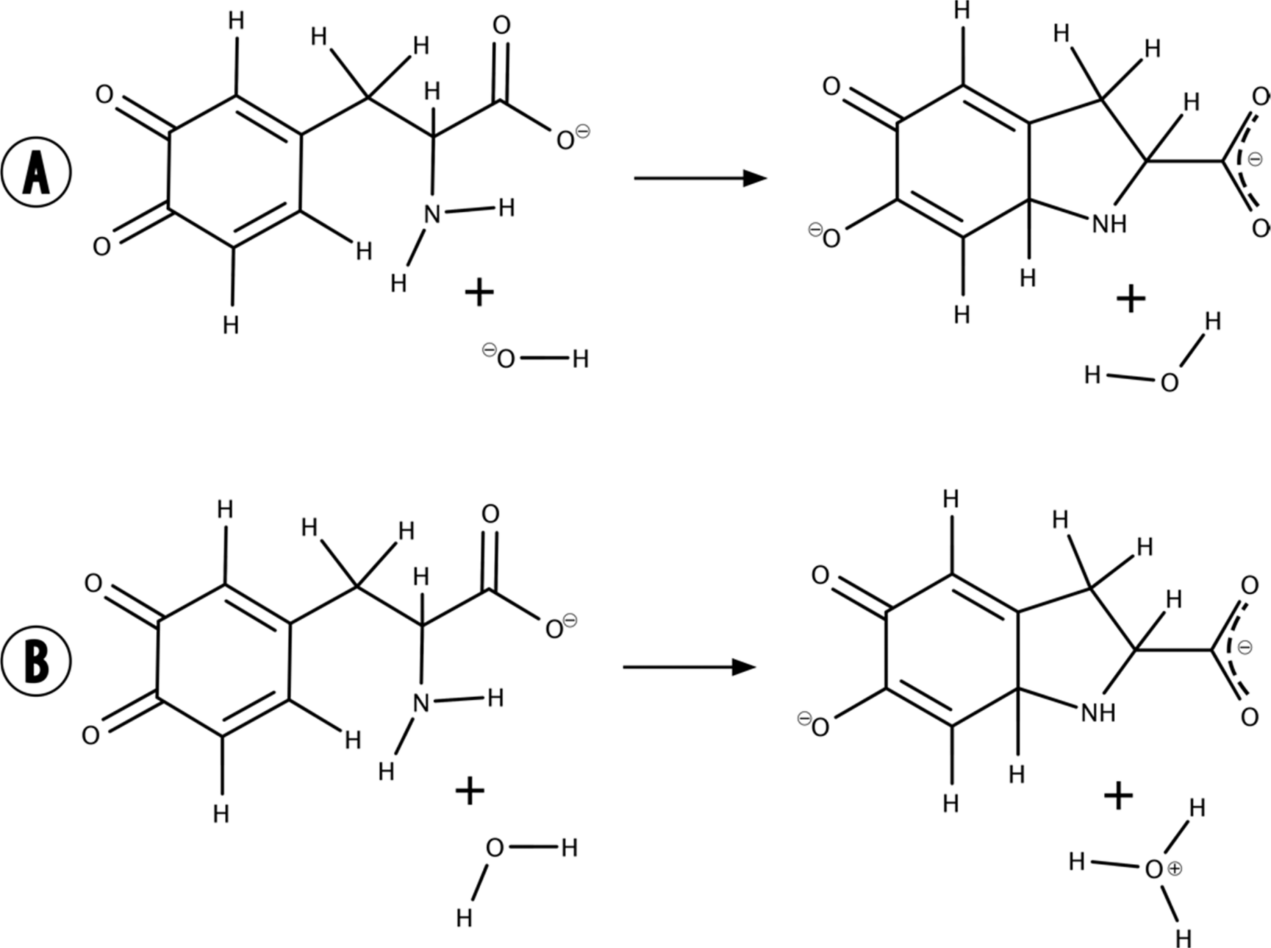
Reaction between (A) dopaquinone and hydroxide ion and (B) dopaquinone and a water molecule.

In the presence of molecular oxygen, dopaquinone tends to enter the redox cycle (like dopamine) and form hydrogen peroxide. The reaction we studied is an intramolecular Michael addition concerted with a proton transfer from the amino group in which L-DOPA is already oxidized to its quinone form dopaquinone (Figure 2).

Since the rate-limiting step requires the presence of a hydroxide ion and a neutral amino group, a strong dependence of the kinetic data on pH is to be expected, as was the case in the dopamine autoxidation reaction,^14^ which was confirmed by Salomäki et al.^15^ Please note that radical mechanism would have no pH dependence. In addition, the presence of divalent and trivalent metal ions catalyzes dopamine autoxidation.^16^

To examine other mechanisms, we also studied the corresponding reaction where the proton acceptor is a water molecule rather than a hydroxide ion. Since the reaction is endergonic, this mechanism is not plausible (*vide infra*).

From Figure 1 it is evident that dopamine and L-DOPA are closely structurally related, therefore the same chemical mechanisms are anticipated for both species. In contrast to L-DOPA, detailed mechanistic studies of dopamine autoxidation are available. Validity of the rate-limiting step for dopamine, consisting of intramolecular Michael addition concerted with proton transfer, is supported by studies reproducing experimental pH-dependent kinetics. For dopamine the cyclization process is rate-limiting, since at pH values below 4.5 intermediates 2,4,5-trihydroxyphenylethylamine and 5-(2-aminoethyl)-2-hydroxy-1,4-benzoquinone start to accumulate.^17^

Our study delves into the mechanism of the rate-limiting step of L-DOPA autoxidation in aqueous solution by applying quantum chemical methods in conjunction with the solvent reaction field and multiscale QM/MM methodology at the Empirical Valence Bond (EVB) level. The Empirical Valence Bond (EVB) approach developed by Warshel is the method of choice for computational enzymology because it is computationally inexpensive and elegantly incorporates the parameters for the reference reaction. In addition to reproducing the experimental kinetics of the reaction in aqueous solution, the obtained EVB parameters are applied in the simulation of the same reaction when L-DOPA is randomly incorporated into the proteins instead of other aromatic amino acids.^10^ Together with additional experimental and computational work, the results presented here contribute to a deeper understanding of L-DOPA side effects induced by the increased oxidative stress and pave the way for improved strategies to treat Parkinson’s disease.

## 2 Computational Details

### 2.1 Experimental Kinetic Data of L-DOPA Autoxidation

The experimental kinetics of L-DOPA autoxidation, in which the rate-limiting step is dopaquinone cyclization, were studied at 37 °C and pH 7.4.^18^ The reported rate constant was 2.56.10^–7^ s^–1^, corresponding to a half-life of 752 h. Utilizing the Eyring-Polanyi equation (eq 5), this gives an experimental activation free energy of 27.55 kcal mol^–1^. Since the rate-limiting step involves a hydroxide ion and a neutral amino group, it is possible to decompose the activation free energy into components associated with the deprotonation of water and the protonated amino group, respectively. The experimental p*K*_a_ value for a water molecule in bulk water is 15.7, while the experimental p*K*_a_ value for the L-DOPA amino group is 8.11^18^ (the *k*_B_*T* value at 37 °C is 0.617 kcal mol^–1^). The free energy associated with the formation of the hydroxide ion can be calculated as follows
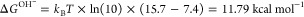
1

and the deprotonation of the amino group as
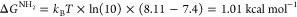
2

Therefore, if both a deprotonated amino group and a hydroxide ion are available, the barrier for the reaction would be much lower, i.e. 27.55 – 11.79 – 1.01 kcal mol^–1^ = 14.75 kcal mol^–1^. It is important to note that the experimental p*K*_a_ value for the amino group is only available for L-DOPA and not for dopaquinone.

### 2.2 Quantum Chemical Calculations

For the quantum chemical analysis of the studied reaction, we performed density functional theory (DFT) calculations using the M06-2X functional (developed by Truhlar and co-workers), which has been shown to be accurate for the calculation of barriers for organic reactions,^19^ in conjunction with the 6-31+G(d,p) basis set. Since the studied reaction not only involves two reactive anions with a low gas-phase barrier but is also highly exergonic, we were unable to identify a reactant minimum or saddle point. To solve this problem, we included the solvent reaction field at the Polarizable Continuum Model (PCM)^20^ and SMD^21^ levels and used water as the solvent. The SCRF-calculated activation free energy was 8.54 kcal mol^–1^, with a reaction free energy of −31.07 kcal mol^–1^. The corresponding SMD values were 10.80 and −26.76 kcal mol^–1^, respectively. Vibrational analysis of both the SCRF and SMD stationary points in the harmonic approximation revealed real frequencies for reactant and product minima, while the transition state had one imaginary frequency indicating the reactive event.

The gas phase activation and reaction energies were determined by single point calculations for the SCRF and SMD geometries. The SCRF-calculated gas phase activation energy was 1.28 kcal mol^–1^ and the reaction energy was −47.04 kcal mol^–1^. The corresponding SMD values were 2.12 and −51.36 kcal mol^–1^, respectively. The SCRF values for the gas phase were used to construct the EVB surface (*vide infra*). The choice of M06-2*X*/6-31+G(d,p)//SCRF M06-2*X*/6-31+G(d,p) was based on our previous good experience with this level of theory in dopamine autoxidation calculations.^14^

We examined one additional mechanism where the hydroxide ion is replaced by a water molecule. Calculations were performed on the SMD M06-2*X*/6-31+G(d,p) level. The transition state was not located despite several attempts. Since water is a much weaker base than a hydroxide ion, the reaction is endergonic with a reaction free energy of 13.54 kcal/mol, therefore one can safely conclude that this mechanism is not plausible.

The computed reaction energy and barrier were used in the calibration of the EVB protocol (*vide infra*) in accordance with standard practice.^22^ The values used for EVB fitting were a gas-phase barrier of Δ*G*_gas_^‡^ = 1.28 kcal mol^–1^ and a reaction energy of Δ_r_*G*_gas_ = −47.04 kcal mol^–1^.

### 2.3 Empirical Valence Bond Calculations

**Figure 3:**
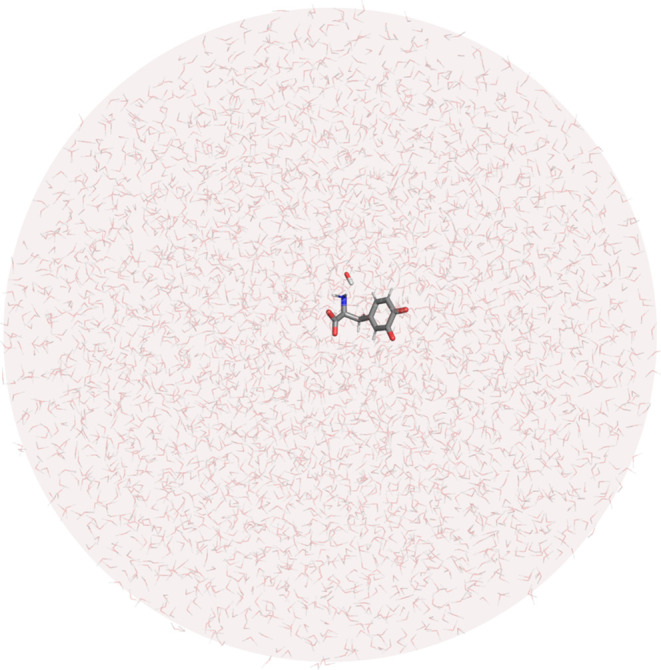
Structure of dopaquinone and hydroxide ion embedded in water, both represented using colored sticks.

The structures of dopaquinone and the adjacent hydroxide ion were constructed using Molden.^23^ Two EVB states were used: the first representing the reacting complex and the second the intermediate state in which the hydroxide ion accepts the proton from the amino group and forms a water molecule, where the cyclization process is concerted with the proton transfer. The atomic charges for both EVB states were calculated by fitting to the electrostatic potential calculated at the HF/6-31G(d) level of theory according to the RESP scheme. The reactive complex was solvated in a spherical cell with a radius of 30 Å centered on the nitrogen atom of L-DOPA and comprising a total of 3778 water molecules, all described with the OPLS-AA force field.^24^ The structure of the reactive complex surrounded by a sphere of water is shown in Figure 3.

All simulations and free-energy calculations were carried out using the *Q5* software package.^25^ The simulations were first performed in the gas phase, followed by simulations in aqueous solution. Soft harmonic position restraints with a force constant of 0.1 kcal mol^–1^ Å^–2^ were applied to all solute atoms. In addition, position restraints with a force constant of 50 kcal mol^–1^ Å^–2^ were applied to three consequent atoms of the aromatic ring to prevent excessive rotational temperature. For the O–N atom pair that is part of the EVB description of the system (i.e., the oxygen atom of the hydroxide ion and the nitrogen atom of L-DOPA), the repulsive part of the Lennard-Jones potential was replaced by Buckingham-type repulsion. Since the reactant state of the simulated system is formally composed of two anions, it dissociates immediately in the gas phase. Therefore, a flat bottom harmonic distance restraint was applied to the hydroxide atom oxygen and amino group nitrogen atom. For all O–N distances between 3.0 and 3.05 Å (where the value of the potential is zero), a force constant of 10 kcal mol^–1^ Å^–2^ was used. Note that the DFT-calculated O–N distance is 3.0 Å. The application of restraints required thorough testing (see Supporting Information, Table S1), reflecting the challenging nature of the simulated system in terms of long-range electrostatics and hydration effects.

**Figure 4:**
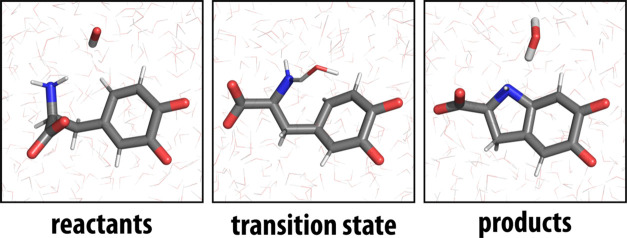
Structures of reactants (left), transition state (center) and products (right) of the reaction. The time-averaged distances between the reacting N atom of dopaquinone and the O atom of the hydroxide are 3.64 ± 0.23, 2.57 ± 0.11, and 3.40 ± 0.19 Å for the reactants, the transition state and the products, respectively.

The production part of both simulations was conducted at 310 K (37 °C) to allow critical comparison with experimental kinetic data. The gas phase simulations were performed at 310 K, while the simulations in solution required a more cautious approach. Initially, the system was equilibrated by gradually increasing the temperature from 1 to 310 K, while simultaneously extending the integration time-step from 0.1 to 1 fs and gradually releasing the applied positional restraints to the values described above. The classical molecular dynamics trajectories for the reaction step were generated using a mapping potential^26,27^ of type
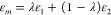
3

where the force field of the reactants (ε_1_) was gradually transformed into that of the products (ε_2_) via the coupling parameter λ. To improve the statistics, we simulated a total of ten replicas using 51 λ-frames, each 0.1 ns long, resulting in 51 ns of MD. The same protocol was employed in our previous work.^28^ Production runs were performed at a temperature of 310 K with a time-step of 1 fs. A spherical cutoff of 10 Å was used for the interactions between dopaquinone, hydroxide ion–water and water–water, while the local reaction field was employed for long-range interactions beyond 10 Å. The structure of the reactive system (corresponding to the reactants, transition state and products) is illustrated in Figure 4 and clearly shows the proton transfer from the amino group to the hydroxide ion that accompanies the cyclization. See also Figure 1.

The corresponding free energy profiles were computed from these simulations using the well-established Free Energy Perturbation/Umbrella Sampling (FEP/US) approach,^26,27,29^ as described in our previous work.^30,31^

The reaction was simulated in the gas phase and in the aqueous solution. The free energy profile in the gas phase was fitted to the quantum chemically calculated barrier height of Δ*G*_gas_^‡^ = 1.28 kcal mol^–1^ and the reaction energy of Δ_r_*G*_gas_ = −47.04 kcal mol^–1^. The mapping yielded calibrated EVB parameters, namely the off-diagonal matrix element *H_ij_* of 96.86 kcal mol^–1^ and the gas phase shift α of 54.72 kcal mol^–1^, which were used for the reaction in water. The trajectories were visualized using the VMD program.^32^

## 3 Results and Discussion

### 3.1 Activation Free Energies Calculated on the EVB Level

**Figure 5:**
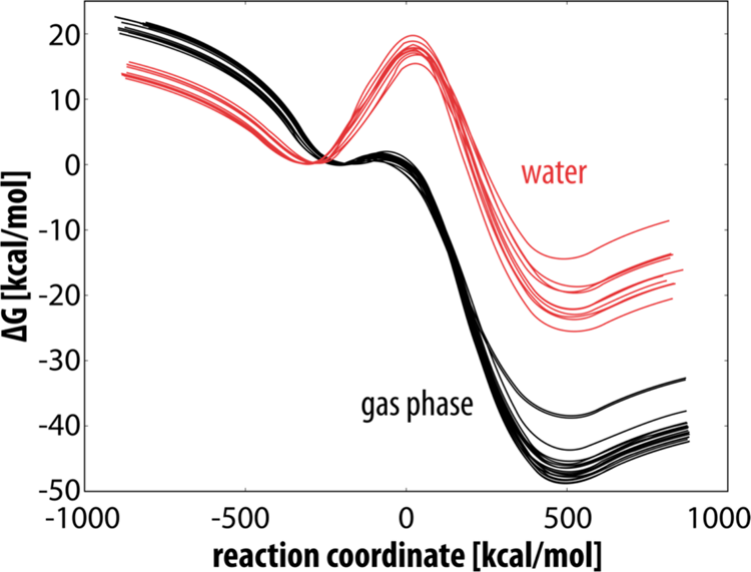
Reaction profiles for the rate-limiting step of dopaquinone decomposition. The gas phase profiles are depicted in black, while the water profiles are in red. The reaction coordinate is defined as the energy difference between EVB states 2 and 1 and is commonly used in displaying EVB free energy profiles.

The reaction profiles for the reaction in the gas phase and in aqueous solution are shown in Figure 5.

The EVB-calculated barrier for the rate-limiting step of dopaquinone autoxidation in aqueous solution involving a hydroxide ion is 18.13 ± 1.12 kcal mol^–1^. The correction for the formation of a hydroxide ion at a pH of 7.4 is Δ*G*^OH–^ = *k*_B_*T* × ln(10) × (15.7–7.4) = 11.79 kcal mol^–1^, while the deprotonation of the amino group at the same pH is estimated to be Δ*G*^NH2^ = *k*_B_*T* × ln(10) × (8.11–7.4) = 1.01 kcal mol^–1^. The computed free energy of activation (calculated by summing these three values) is 30.93 ± 1.12 kcal mol^–1^, which is in good agreement with the experimental value of 27.55 kcal mol^–1^. The experimental value for the reaction free energy is not available, while the calculated reaction free energy is exergonic, which is in agreement with the proposed reaction mechanism.

The free energy cost for the formation of the hydroxide ion is comparable to the rest of the chemical step, which involves cyclization and the formation of a water molecule. Note that during the chemical step, the hydroxide ion is converted to a neutral water molecule and doubly charged dopaquinone is formed. It should be emphasized that a critical element that determines the accuracy of the simulation are the nonbonding parameters between these two anions and water. Various experimental values for the free energies of hydration of hydroxide ion have been reported, ranging from −90.6 to −110.0 kcal mol^−1^,^33^ with the most accurate value being −106.4 ± 0.5 (kcal mol^–1^).^34^ An additional source of error is the hydration free energy of negatively charged dopaquinone with the net charge of −1 for reactants and the corresponding double charged anion for the products. In contrast to hydroxide ion and water, experimental hydration free energies for these two species are not available. Truhlar and co-workers estimated that the error bar of calculated hydration free energies of ionic species is around 4 kcal mol^–1^, which is consistent with our results.^21,35^ Based on these facts, the slight discrepancy between the experimental and calculated activation free energies in our work is not surprising.

### 3.2 Activation Free Energies Calculated by Solvent Reaction Field

We were not able to quantitatively reproduce the activation energy with the solvent reaction field approach. Using the PCM solvation model (in conjunction with the M062*X*/6-31+G(d,p) level of theory), we calculated an activation energy of 8.54 kcal mol^–1^, while the energy calculated using the SMD solvation model was 10.80 kcal mol^–1^. After correcting these values for the formation of hydroxide ion and the deprotonation of the charged amino group, the adjusted activation energies are 21.34 and 23.60 kcal mol^–1^ for the PCM and SMD models, respectively. These two values underestimate the experimental barrier of 27.55 kcal mol^–1^, but the SMD results are closer to the experimental barrier, reflecting the very careful parametrization of this model. We have had very good experience with the SMD solvent reaction field because, unlike all other solvent reaction field models, only SMD can reproduce the octanol/water partition coefficients for the neutral and charged form of local anesthetics.^36^

This is further evidence that the studied reaction is extremely demanding in terms of hydration description and is a very stringent test of the quality of the solvation models applied.

### 3.3 pH Dependence of Reaction Kinetics

The proposed mechanism of the dopaquinone reaction with a hydroxide ion allows us to express analytically the activation free energy as a function of the pH of the solution by using the following eq (eq 4). We consider the experimental activation energy of the reaction when hydroxide and a neutral amino group are present. Since we are only interested in the pH values close to the physiological state, the acidic carboxyl group (p*K*_a_ = 2.3) will be deprotonated throughout this range.
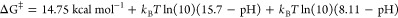
4

Note that at acidic pH, the free energy cost for deprotonation of the hydroxide ion and charged amino group is much higher than at neutral pH. This expression can then be used in the Eyring-Polanyi equation, which links the activation energy Δ*G*^⧧^ to the reaction rate constant *k*_rate_.
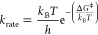
5This results in the following pH dependence:
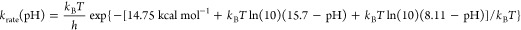
6from which we can then derive the following:
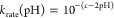
7where the constant *c* represents all quantities not dependent on the pH.

**Figure 6:**
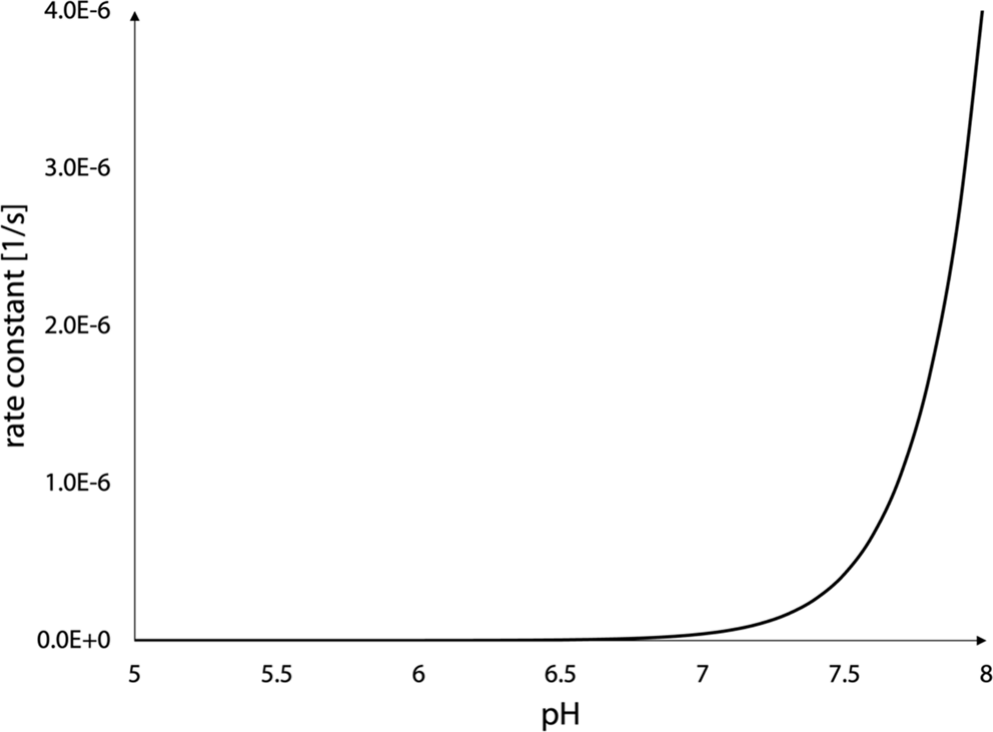
pH-dependent rate constant for dopaquinone decomposition.

The analytical expression for the pH-dependent rate constant of L-DOPA autoxidation has the same functional form as that of dopamine autoxidation,^14^ but the dopamine reaction is much faster. Moreover, eq 6 agrees with the experimental pH-dependent rate constants,^18^ although the experiments were performed only for pH values of 7.4 and 2. The latter value is physiologically irrelevant and the protonation state of the carboxyl group with a p*K*_a_ of 2.31 would also be different. The derived dependence of the rate constant on the pH of the solution is shown in Figure 6.

## 4 Conclusions

We have investigated the reaction mechanism of L-DOPA autoxidation using quantum chemical methods and have shown that at physiological pH the rate-limiting step is the formation of a hydroxide ion that reacts with the L-DOPA metabolite dopaquinone. An analytical way to describe the pH dependence of the rate constant was derived and the proposed model agrees with the experimental data. A strongly pH-dependent rate constant supports the assumption that the rate-limiting step involves a heterolytic reaction and that a reaction mechanism involving free radicals is not plausible under physiological conditions. By using multiscale QM/MM simulations at the Empirical Valence Bond level, we obtained an activation free energy of 30.93 kcal mol^–1^, which is in reasonable agreement with the experimental value of 27.55 kcal mol^–1^. Obviously, direct dopaquinone oxidation with the experimental rate constant of 2.56.10^–7^ s^–1^ and a half-life of 752 h at 37 °C and pH 7.4 is much slower than the corresponding reaction involving dopamine-*o*-quinone with a rate constant of 0.147 s^–1^ and a half-life of 4.7 s. Therefore, it is anticipated that the reaction in which L-DOPA is directly autoxidized is less relevant and that it is more efficient for L-DOPA to be decarboxylated first, resulting in dopamine, which is autoxidized much faster. Furthermore, the strong dependence of the rate constant on the pH of the solution implies that the dopaquinone reaction does not occur in the acidic compartments of the neuron (e.g., the vesicles with a pH of 5.9), which is also true for dopamine-*o*-quinone.^14,37^ A challenge for the future remains experimental and computational examination of deuteration effects on L-DOPA. Typically, H/D kinetic isotope effects for reactions in solution are between 3 and 8, which implies substantially prolonged elimination time. Please note that the first deuterated drugs are already in clinical practice.^38^ A suitable computational method for the exploration of kinetic isotope effects is path integration, which we successfully employed in our previous studies.^39,40^

The fact that L-DOPA is indiscriminately incorporated into all central nervous system proteins instead of aromatic amino acids strongly suggests that these altered proteins serve as a consistent and permanent source of reactive oxygen species in Parkinson’s patients treated with L-DOPA. It remains unclear whether the protein environment is catalytic or anticatalytic in this process. We performed a very preliminary calculation of the reaction where L-DOPA is built into a protein by considering four residues of monoamine oxidase B by using a quantum chemical calculations cluster model and solvent reaction field for the rest of the protein. The calculated barrier is slightly lower than for the reaction without the protein environment. Changed reactivity can be rationalized by changed nucleophilicity of the nitrogen atom when amino group is changed to amide group and changed solvation effects relative to water.

The Empirical Valence Bond parameters that we derived in this study together with the postulated reaction mechanism could serve as a reference reaction for various protein environments. Comprehensive research efforts, including sequencing, structural analysis, kinetic studies, and clinical observations, are crucial to understand the side effects of L-DOPA and ideally to improve the pharmacological management of Parkinson’s disease. Our study represents an initial computational effort in this direction and lays the foundation for future investigations.

## Supporting Information Available

Calculated activation free energy Δ*G*^‡^ and reaction free energy Δ*G*_R_ in kcal mol^–1^ for the reaction in the gas phase and aqueous solution depending on the simulation parameters (PDF)
