# Supplementary material for: L-DOPA Autoxidation: An Empirical Valence Bond Simulation of the Reactive Step
Source: J Phys Chem B. 2024 Aug 24;128(35):8355–61. doi: 10.1021/acs.jpcb.4c03002 (PMC11382278; doi:10.1021/acs.jpcb.4c03002)
Supplement: Supplementary file 1 — jp4c03002_si_001.pdf [file jp4c03002_si_001.pdf]

Supporting Information

L-DOPA Autoxidation: An Empirical Valence Bond Simulation of the Reactive Step

Alja Prah[1,2] *and Janez Mavri*[1,*]

1 Laboratory for Computational Biochemistry and Drug Design, National Institute of Chemistry, Ljubljana, Slovenia

2 Networking Infrastructure Centre, Jožef Stefan Institute, Ljubljana, Slovenia

* email: janez.mavri@ki.si

Table S1. Calculated activation free energy $\Delta G^{\ddagger}$ and reaction free energy ΔG_R in kcal mol^{-1} for the reaction in the gas phase and aqueous solution depending on the simulation parameters. Please note that for aqueous solution it is necessary to add the free energy cost for formation of hydroxide ion of 11.79 kcal mol^{-1} and deprotonation of the amino group of 1.01 kcal mol^{-1} at pH value of 7.4. Standard deviations are calculated from the values of ten replicas. Thermodynamic perturbation was performed either forward (λ was perturbed from 0 to 1, *i.e.* from the reactants to the products) or backward (λ was perturbed from 1 to 0, *i.e.* from the products to the reactants). K_{ij} represents the harmonic force constant in kcal mol^{-1} Å^{-2} for the position restrains for all EVB atoms. Flat bottom potential is applied for the hydroxide oxygen atom – nitrogen atom distances either for EVB state one (1), EVB state two (2) or both (0). The nitrogen and oxygen atom are held together by a flat bottom harmonic well potential, which is zero between 3.0 and 3.05 Å and has a force constant of 10.0 kcal mol^{-1} Å^{-2} for other distances. It is active in both FEP states. Values corresponding to the bolded row are reported in the article.

Direction	K_{ij}	Flat bottom potential	$\Delta G^{\ddagger}$	ΔG_R
**backward water**	**0.5**	**N O 3.0 3.05 10.0 0**	**18.13 ± 1.12**	**-22.02 ± 2.24**

forward water	0.5	N O 3.0 3.05 10.0 0	23.29 ± 3.64	-20.58 ± 3.34
forward water	3.0	N O 3.0 3.05 10.0 0	25.26 ± 2.76	-21.58 ± 2.37
**backward gas**	**0.5**	**N O 3.0 3.05 10.0 0**	**1.28 ± 0.38**	**-47.04 ± 1.36**
forward gas	3.0	N O 3.0 3.05 10.0 0	1.28 ± 0.87	-47.04 ± 2.76
